# Lignocellulose conversion of ensiled *Caragana korshinskii* Kom. facilitated by *Pediococcus acidilactici* and cellulases

**DOI:** 10.1111/1751-7915.14130

**Published:** 2022-08-12

**Authors:** Yixin Zhang, Musen Wang, Samaila Usman, Fuhou Li, Jie Bai, Jiayao Zhang, Xusheng Guo

**Affiliations:** ^1^ State Key Laboratory of Grassland Agro‐Ecosystems, School of Life Sciences Lanzhou University Lanzhou PR China; ^2^ Probiotics and Biological Feed Research Centre Lanzhou University Lanzhou PR China; ^3^ State Key Laboratory of Grassland Agro‐Ecosystems, College of Pastoral Agriculture Science and Technology Lanzhou University Lanzhou PR China

## Abstract

To explore the biofuel production potential of *Caragana korshinskii* Kom., *Pediococcus acidilactici* and an exogenous fibrolytic enzyme were employed to investigate the fermentation profile, structural carbohydrates degradation, enzymatic saccharification and the dynamics of bacterial community of *C*. *korshinskii* silage. After 60 d of ensiling, all additives increased the fermentation quality. The highest lactic and acetic acids and lowest non‐protein nitrogen (NPN) and ammonia nitrogen (NH_3_‐N) were observed in *P. acidilactici* and *Acremonium* cellulase (PA + AC) treated silage. Additionally, all additives significantly increased the ferulic acid content and fibre degradability with the highest values obtained from PA + AC silage. The bacterial community in all silages was dominated by *P. acidilactici* throughout the entire fermentation process. The bacterial community was also modified by the silage additives exhibiting a relatively simple network of bacterial interaction characterized by a lower bacterial diversity in *P. acidilactici* (PA) treated silage. The highest 6‐phospho‐beta‐glucosidase abundance was observed in PA‐treated silage at the mid‐later stage of ensiling. PA treatment exhibited lower structural carbohydrates degradation but performed better in lignocellulose conversion during enzymatic saccharification. These results indicated that pretreating *C. korshinskii* improved its silage quality and potential use as a lignocellulosic feedstock for the production of bio‐product and biofuel.

## Graphical abstract

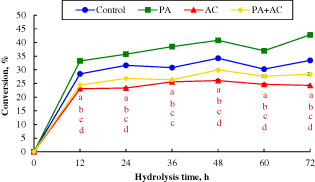

## INTRODUCTION

Energy security has become a dominant issue for developing countries due to increasing global warming and climate change (Alper & Stephanopoulos, 2009; Ranjan & Moholkar, 2012). Renewable energy resources such as crop straws, agricultural residues and energy plants, are widely used in addressing environmental challenges and fuel production (Abraham et al., 2020). Lignocellulosic biomass as the most abundant renewable energy resource help to circumvent the competition with cereals in biofuel production (Birgen et al., 2021). *Caragana korshinskii* Kom., a forage shrub species for vegetation rehabilitation, is widely planted in the arid and semi‐arid regions of Asia and Europe for prevention and control of desertification as well as ecological restoration. However, to enable fresh regrowth and sustainability of *C. korshinskii* plantation, old stems are being pruned and cut back frequently which annually generates over 4 million tons of stubbles in China (Li et al., 2021). In some studies, it was found that *C. korshinskii* exhibited great potential for silage making and as a feedstock for biofuel production (Li et al., 2021; Zhang et al., 2009). However, the utilization efficiency of *C. korshinskii* is not always satisfactory, owing to its high lignin content and complex fibre structure (Xu et al., 2006). In addition, *C. korshinskii* also has storage and biotransformation difficulty, thereby requiring efficient preservation and pretreatment methods to achieve year‐round utilization.

Microbial anaerobic fermentation is considered an effective pretreatment method employed for the preservation, and subsequent supply of high‐quality feedstock for animals and biofuel production (Alper & Stephanopoulos, 2009; Liu et al., 2019). However, *C. korshinskii* is difficult to be ensiled due to its higher buffering capacity and crude fibre content (Ke et al., 2017; Li et al., 2021). Meanwhile, ingestion, digestion and absorption of *C. korshinskii* are restricted for animals due to the presence of thorns and other secondary metabolites of the shrub (Jurado et al., 2009; Li, Han, et al., 2020). Preserving *C. korshinskii* as silage can conserve its nutrients and soften the stipule thorn, thereby improving its palatability (Zhang et al., 2009). Therefore, it is necessary to explore effective methods to break the fibrous structure of *C. korshinskii*. Various silage inoculants and exogenous cellulases have been applied to improve fermentation quality by accelerating acidolysis or enzymolysis to decrease the structural complexity of plant cell walls (Khota et al., 2016; Li et al., 2018; Liu et al., 2019). Many additives, such as molasses, organic acid and fibrolytic enzyme, have been used in pretreating lignocellulosic materials to preserve the nutrients and enhance fibre degradation (Desta et al., 2016; Liu et al., 2019). Several studies have explored the application of different lactic acid bacteria (LAB) and cellulose enzymes to enhance fibre degradation and improve the ruminal digestibility of silages (Khota et al., 2016; Li, Ke, et al., 2020). Compared with other bacterial strains, *Pediococcus acidilactici*, a homofermentative LAB, can grow rapidly and produce lactic acid in high pH media to inhibit spoilage organisms, thereby improving fermentation quality and enhancing fibre degradability (Alhaag et al., 2019; Porto et al., 2017; Zhang et al., 2020). Hence, the sole application of *P. acidilactici* in silages can initiate rapid fermentation at the early stage of ensiling (Bai et al., 2021; Yang et al., 2019), while the combination of *P. acidilactici* and cellulase could be an effective way to improve both fermentation quality and fibre degradability. We hypothesized that application of a rapid start‐up LAB alone, or in combination with cellulase before ensiling could improve fermentation quality and/or promote fibre degradability as well as reduce nutrients loss from the ensiled forage. To our best knowledge, however, far less seems to be known about the effects of *P. acidilactici* and cellulase on the fermentation profile, fibre degradation and bacterial community of *C. korshinskii* silage. Therefore, the objective of this study was to evaluate the fermentation profile, structural carbohydrates degradation and enzymatic saccharification of *C. Korshinskii* silage treated with a rapid start‐up *Pediococcus acidilactici* strain and an exogenous cellulose enzyme.

## EXPERIMENTAL PROCEDURES

### Feedstocks and silage additives

Branches (with leaves and pods) of *C. korshinskii* were manually harvested at the podding stage from Yuzhong County, Gansu Province, China (35°85′N, 104°12′E) on 19th June 2020. *P. acidilactici* and *Acremonium* cellulase (AC, Meiji Seika Pharma Co., Ltd, Tokyo, Japan) were supplied as freeze‐dried powders and applied as silage additives. The *P. acidilactici* bacterial strain was isolated from corn stalk silage and stored in our laboratory. The viable count of *P. acidilactici* powder was 2.5 × 10^11^ colony‐forming units (CFU) per gram. Based on the manufacturer's description, *Acremonium* cellulase activity was more than 1000 U/g, and the patent formula of plant cell wall‐degrading enzymes was composed of *β*‐Glucanase, *α*‐Arazyme, *α*‐Galactosidase, *β*‐Galactosidase and *β*‐Xylanase. Before the onset of the experiment, *P. acidilactici* and *Acremonium* cellulase were stored at 4°C.

### Silage preparation

Fresh *C. korshinskii* was taken to a laboratory and chopped into 2 to 3 cm segments using a manual forage chopper (F80221; Wuyang County Mengba department store, Linyi, China). Subsequently, *C. korshinskii* was mixed into a pile and randomly separated into 84 sub‐samples (every sub‐sample weighted approximately 400 g). Four random fresh sub‐samples were collected and frozen at −20°C pending further analysis. The remaining 80 sub‐samples (4 treatments × 5 time points × 4 replicates) were randomly subject to the following treatments: (1) distilled water (Control); (2) *P. acidilactici* (PA); (3) *Acremonium* cellulase (AC); (4) a combination of *P. acidilactici* and *Acremonium* cellulase (PA + AC). The application rate of *P. acidilactici* was 1 × 10^5^ CFU/g fresh weight (FW). The application rate of commercial cellulase was 0.3 g/kg FW as described by Li, Ke, et al. (2020). To evenly apply the additives to the chopped forages, each additive was diluted with sterile distilled water (10 ml/kg FW). For the Control, the same volume of sterile distilled water was applied. After thorough mixing, all the treated samples were ensiled in mini‐silo bags (280 mm × 320 mm; Cangzhou Hualiang Packaging Co. Ltd., Hebei, China) and vacuum‐sealed using a vacuum sealing machine (DZ400/ZT, Wenzhou Overseas Chinese Packaging machinery factory, Zhejiang, China). The mini‐silos were fermented for 3, 7, 14, 30 and 60 d at room temperature of 25 ± 2°C.

### Chemical and ferulic acid analyses

To determine the fermentation parameters, a sample (20 g) from each silo was squeezed with 180 ml of distilled water in a high‐speed blender, then filtered through four layers of medical gauze. The filtrate was divided into two portions. The first portion was acidized to a pH of around 2.0 by H_2_SO_4_ (7.14 M) immediately after measuring the silage pH. The acidized liquid was filtered through a 0.22‐μm filter for the determination of organic acids (lactic, acetic, propionic and butyric acids) according to the method of Zhang et al. (2021). Another portion of the filtrate was mixed with trichloroacetic acid (25%, w/v) at a ratio of 4:1 (v/v) and stood for 1 h at room temperature to depose the true protein. Subsequently, after 15 min of centrifugation at 18,000*g* at 4°C, the supernatant was analysed for NH_3_‐N and WSC following the procedure of Thomas (1977), and NPN was measured as described by Licitra et al. (1996).

To measure the DM content, fresh and silage samples were dried for 72 h at 65°C by using a thermostatic oven dryer (Y101A‐4, Changzhou Depu Textile Technology Co. Ltd., Jiangsu, China) and ground to pass through a 1‐mm sieve for nutrient analyses according to AOAC (2005). Total nitrogen was determined by the Automatic Kjeldahl apparatus (K9840, Hannon instrument Co. Ltd., Jinan, China), and CP was estimated as total nitrogen × 6.25. The contents of aNDF, ADF and ADL were measured according to the methods of Robertson and Van Soest (1981) using a fibre analyser (A2000I, Ankom Technology, Fairport, NY). Heat‐stable *α*‐amylase was used during aNDF analysis. Hemicellulose and cellulose contents were calculated in line with Li, Ke, et al. (2020). Ferulic acid was extracted and determined as described in the method of Zhao et al. (2014).

### Bacterial community composition SMRT analysis

Silage bacterial community at the species level was determined as comprehensively reported by Bai et al. (2021) and Wang et al. (2021). In brief, the bacterial profile was revealed at species level throughout the single‐molecule real‐time sequencing technology (SMRT). Total bacteria DNA extraction of fresh and ensiled *C. korshinskii* was performed with a TIANamp Bacteria DNA Kit (DP302‐02, Tiangen Biotech Co., Ltd. Beijing, China) according to the manufacturer's protocol. The concentration of extracted DNA samples was determined using a Thermo Fisher NanoDrop instrument (ND‐2000, United States). The PCR amplification of the bacterial 16S rRNA gene was carried out by using primers 27F (5′‐GRGTTYGATYMTGGCTCAG‐3′) and 1492R (5′‐RGYTACCTTGTTACGACTT‐3′). The bacterial 16S amplification program was conducted following the description of Zhou et al. (2020). Sequencing of the amplicons and data analysis was carried out on a PacBio platform (Pacific Biosciences, Menlo Park, CA, United States) by the company of Wuhan Frasergen Bioinformatics Co., Ltd. (Wuhan sector, China, in the year 2020). Microbial networks were used to statistically calculate; meanwhile, the identification of keystone taxa in the microbial communities was performed by the combined score of low betweenness centrality, high closeness centrality and high mean degree (Bai et al., 2021). Microbial functions were proof‐checked from the KEGG database using PICRUSt.

### Enzymatic saccharification of silages

The enzymatic saccharification of *C. Korshinskii* silage was conducted after 60 days of ensiling using the Laboratory Analytical Procedure of the National Renewable Energy Laboratory (Resch et al., 2015) with minor modification. Freeze‐dried sample (0.3 g on DM basis) was weighed and transferred in 5 ml sodium citrate buffer (0.1 M, pH 5.0) which contained *Acremonium* cellulase (1 mg/ml) based on cellulose content. A 200 μl of 2% sodium azide solution was added to avoid microbial contamination. Distilled water was supplemented to bring the total volume to 10 ml before the incubation. Another tube was used as a substrate blank with the same dried biomass, the same volume of buffer, antimicrobial agent and distilled water. An enzyme blank was also prepared in another tube with buffer, azide solution, water and enzyme solution. All tubes were incubated in a constant temperature shaker for 72 h at 50°C and 160 rpm. At each 12 h interval, samples were collected and the reaction was terminated at 100°C for 10 min. Glucose and xylose contents were determined by an Agilent high‐performance liquid chromatography 1200 (Agilent Technologies, Inc., Germany; column: Carbomix H‐NP10, Sepax Technologies, United States; detector: Refractive Index Detector, Agilent Technologies, Inc., Germany; eluent: 0.6 ml/min, 2.5 mM H_2_SO_4_; temperature: 55°C) after centrifugation at 10,000*g* for 10 min and filtered through a 0.22‐μm filter membrane. The cellulose conversion was calculated following the Laboratory Analytical Procedure of the National Renewable Energy Laboratory of Selig et al. (2008), and the formula is as follows:

$$\text{Cellulose conversion}\;\left(\%\right)=\frac{\text{glucose yield}\times 0.9}{\text{cellulose}\%}\times 100$$

### Statistical analysis

All data on fermentation parameters and chemical composition at the end of ensiling (60 d) as well as level 2 KEGG orthologue gene and 6‐phospho‐beta‐glucosidase abundance within the same ensiling time were analysed by one‐way ANOVA (SPSS 21.0, Inc., Chicago, IL, United States). Significant differences among means (*p* < 0.05) were declared by Tukey's honestly significant difference (HSD) test.

General linear model (GLM) procedure was used to analyse the bacterial diversity according to 4 × 5 factorial experiment model: *Y*_*ij*_ = *μ* + *E*_*i*_ + *T*_*j*_ + (*E* × *T*)_*ij*_ + *e*_*ij*_, where *Y*_*ij*_ = response variable; *μ* = overall mean; *E*_*i*_ = effect of the ensiling time (*i* = 1, 2, 3, 4, 5); *T*_*j*_ = effect of treatment (*j* = 1, 2, 3, 4); (*E* × *T*)_*ij*_ = effect of interaction between the ensiling time and treatment and *e*_*ij*_ was the residual error.

The non‐structural carbohydrate content obtained after enzymatic saccharification was also analysed using GLM procedure according to 4 × 6 factorial experiment model: *Y*_*ij*_ = *μ* + *H*_*i*_ + *T*_*j*_ + (*H* × *T*)_*ij*_ + *e*_*ij*_, where *Y*_*ij*_ = response variable; *μ* = overall mean; *E*_*i*_ = effect of the hydrolysis time (*i* = 1, 2, 3, 4, 5, 6); *T*_*j*_ = effect of treatment (*j* = 1, 2, 3, 4); (*E* × *T*)_*ij*_ = effect of interaction between the ensiling time and treatment and *e*_*ij*_ was the residual error. Significant differences among means (*p* < 0.05) were declared by Tukey's HSD test.

## RESULTS

### Fermentation and chemical characteristics of *C. korshinskii* before ensiling and ensiled for 60 d

The chemical composition of fresh *C. korshinskii* is shown in Table 1. The fresh *C. korshinskii* exhibited a dry matter (DM) content of 551 ± 7.57 g/kg FW before ensiling, and the pH value was 6.35 ± 0.01. The concentrations of neutral detergent fibre (aNDF), acid detergent fibre (ADF), acid detergent lignin (ADL), hemicellulose and cellulose were quantified before ensiling, which were 335 ± 3.78, 215 ± 3.56, 57.5 ± 1.12, 120 ± 7.34 and 158 ± 2.46 g/kg DM respectively. In addition, ferulic acid concentration was 1091 ± 1.01 mg/kg DM.

**TABLE 1:** Chemical characteristics of freshly chopped *C. korshinskii* before ensiling

Item^a^	Value^b^
DM (g/kg FW)	551 ± 7.57
pH	6.35 ± 0.01
WSC (g/kg DM)	17.3 ± 0.02
CP (g/kg DM)	202 ± 0.50
NPN (g/kg TN)	114 ± 1.62
NH_3_‐N (g/kg TN)	1.53 ± 0.11
aNDF (g/kg DM)	335 ± 3.78
ADF (g/kg DM)	215 ± 3.56
ADL (g/kg DM)	57.5 ± 1.12
Hemicellulose (g/kg DM)	120 ± 7.34
Cellulose (g/kg DM)	158 ± 2.46
Ferulic acid (mg/kg DM)	1091 ± 1.01

^a^ DM, dry matter; FW, fresh weight; WSC, water‐soluble carbohydrates; CP, crude protein; NPN, non‐protein nitrogen; NH_3_‐N, ammonia nitrogen; aNDF, neutral detergent fibre assayed with a heat‐stable amylase and expressed inclusive of residual ash; ADF, acid detergent fibre; ADL, acid detergent lignin.

^b^ Data are presented with means ± standard deviation from three independent experiments.

**FIGURE 1:**
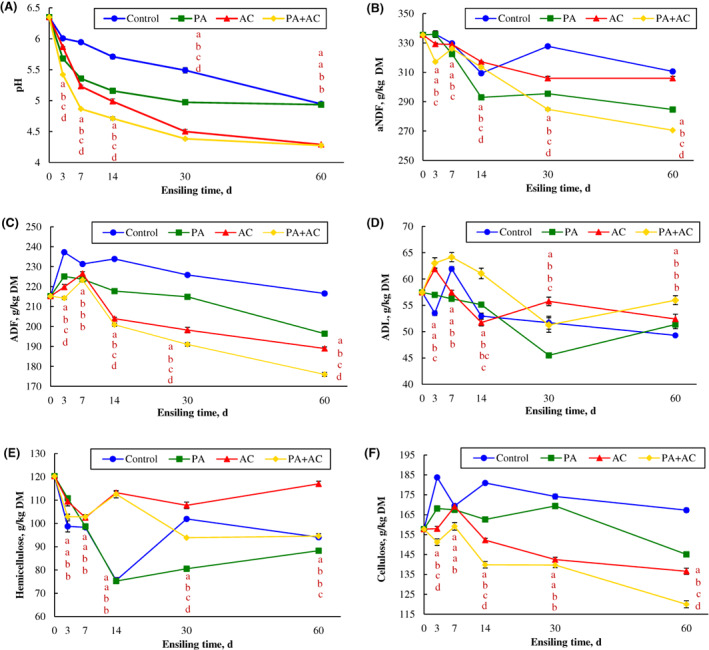
Dynamics of pH (A) and structural carbohydrates (B, aNDF; C, ADF; D, ADL; E, hemicellulose; F, cellulose) of ensiled *C. korshinskii* as influenced by additives and ensiling period. Treatment: Control, without additive; PA, *P. acidilactici*; AC, *Acremonium* cellulase; PA + AC, a combination of *P. acidilactici* and *Acremonium* cellulase; aNDF, neutral detergent fibre assayed with a heat‐stable amylase and expressed inclusive of residual ash; ADF, acid detergent fibre; ADL, acid detergent lignin. Means with different lowercases (a–d) among treatments at the same time point differed (*p* < 0.05; *n* = 4, bars indicate standard error of means).

The fermentation and chemical characteristics of *C. korshinskii* silage are shown in Table 2. There were significant effects of additives on lactic acid, acetic acid and propionic acid contents of the silages. Although all additives enhanced lactic acid and acetic acid contents when compared to the Control, PA + AC treatment resulted in the highest lactic acid and acetic acid contents, with a corresponding lowest propionic acid content (*p* < 0.001). Butyric acid was not detected in any of the treatments. The highest DM content after ensiling was recorded in the PA group, while the DM loss was highest in the Control group. A significant effect (*p* < 0.001) of additives was also observed on the water‐soluble carbohydrates (WSC) of *C. korshinskii* silages in the present study. In contrast to the Control, the addition of AC had the highest WSC content while the sole application of *P. acidilactici* recorded the lowest WSC content. The silages treated with PA and AC had higher crude protein (CP) content compared to the Control silage, but no difference was found between PA + AC and other silages. The lowest contents of non‐protein nitrogen (NPN) and ammonia nitrogen (NH_3_‐N) were observed from silages inoculated with the combination of *P. acidilactici* and *Acremonium* cellulase. The addition of *P. acidilactici* and commercial cellulase (PA‐, AC‐ and PA + AC‐treated silages), as well as the ensiling time, had a significant effect on the silage pH (Figure 1A). The additives decreased the pH values at the initial stage of ensiling (3 d), especially in *P. acidilactici* treatments (PA‐ and PA + AC‐treated silages). Subsequently, the pH values declined continuously with a significant difference among the treatments until 30 d, and the lowest pH value was observed in PA + AC‐treated silage. The pH values of *C. korshinskii* silages at 60 d were all below 4.95, and the addition of AC and PA + AC significantly decreased the pH values compared with the Control and PA groups after 60 d of ensiling.

**TABLE 2:** Fermentation and chemical characteristics of ensiled *C. korshinskii* (60 d) as influenced by additives

Item^a^	Treatment^b^				*SEM* ^c^	*p*‐value
	Control	PA	AC	PA + AC		
Lactic acid (g/kg DM)	32.2^c^	33.9^b^	35.1^b^	42.8^a^	1.067	<0.001
Acetic acid (g/kg DM)	18.9^c^	20.9^b^	21.2^b^	25.3^a^	0.636	<0.001
Propionic acid (g/kg DM)	17.0^a^	11.9^b^	10.1^c^	10.7^c^	0.716	<0.001
DM (g/kg FW)	549^b^	561^a^	540^b^	546^b^	2.207	0.001
DM loss (g/kg DM)	22.5^a^	21.1^ab^	18.8^b^	20.2^ab^	0.307	0.008
WSC (g/kg DM)	0.84^b^	0.58^c^	1.99^a^	0.84^b^	0.008	<0.001
CP (g/kg DM)	204^b^	207^a^	208^a^	206^ab^	0.540	0.006
NPN (g/kg TN)	441^a^	427^b^	418^c^	347^d^	9.355	<0.001
NH_3_‐N (g/kg TN)	31.0^a^	26.9^b^	23.5^c^	21.0^d^	0.974	<0.001

^a^ DM, dry matter; FW, fresh weight; WSC, water‐soluble carbohydrate; CP, crude protein; NPN, non‐protein nitrogen; NH_3_‐N, ammonia nitrogen.

^b^ PA, *P. acidilactici*; AC, *Acremonium* cellulase; PA + PC, a combination of *P. acidilactici* and *Acremonium* cellulose; Means with different letters in the same row (a–d) indicate a significant difference (*p* < 0.05).

^c^ *SEM*, standard error of the mean.

### Dynamics of structural carbohydrates during *C. korshinskii* ensiling process

The effects of additives and ensiling time on the dynamics of aNDF, ADF, ADL, hemicellulose and cellulose of *C. Korshinskii* silage are shown in Figure 1B–F. Throughout the fermentation period, ensiling times and additives contributed to the complexity of the structural carbohydrates' changes. At the early stage of ensiling (3 d), the AC and PA + AC‐treated silages had a conspicuous decrease in aNDF and ADF contents, but an opposite result was observed in ADL content (Figure 1B–D). Compared to Control and other additives treatments, the addition of PA led to a huge reduction in the content of aNDF with advancing ensiling time until 14 d, and a decreasing trend was found in all treatments. As the ensiling time reached the mid‐fermentation (30 d), there was an obvious difference in aNDF among all the treatments up to the end of the ensiling time of our study. However, the application of PA + AC showed the lowest content of aNDF at the end of the fermentation. The effects of *P. acidilactici* on aNDF maintained a plateau from the mid up to the end of the ensiling time, with a lower concentration compared to the Control and AC‐treated silages. The Control silage had the highest ADF content throughout the fermentation period compared to other treatments (*p* < 0.05). No difference was observed in ADF among PA, AC and PA + AC treatments at 7 d, but a marked difference appeared continuously with the advancing period of ensiling. The PA + AC treatment showed the lowest content of ADF from 14 to 60 d of fermentation. The higher content of ADL was obtained after the addition of *P. acidilactici* combined with *Acremonium* cellulase throughout the ensiling process except for the 30 d, and the trend was comparable with the group of PA which declined to the lowest at 30 d. With the advancement in the fermentation period, differences among treatments were obtained in hemicellulose and cellulose contents (Figure 1E,F). At the initial stage of ensiling, PA and AC‐treated silages had a higher content of hemicellulose, subsequently, AC and PA + AC groups showed higher content of hemicellulose than Control and PA treatments from 7 to 14 d. At 30 d, the hemicellulose content was significantly different among the four treatments. The PA‐treated silage had the lowest hemicellulose content than other treatments after 30 and 60 d of ensiling. In addition, the trend in cellulose content was similar to that of the ADF.

### Concentration of ferulic acid during *C. korshinskii* ensiling period

**FIGURE 2:**
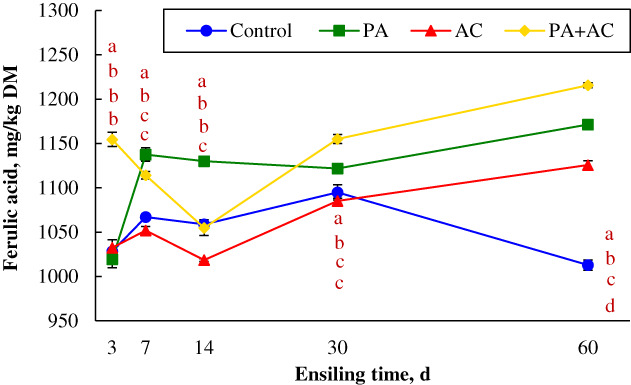
Ferulic acid concentrations of ensiled *C. korshinskii* as influenced by additives and ensiling period. Treatment: Control, without additive; PA, *P. acidilactici*; AC, *Acremonium* cellulase; PA + AC, a combination of *P. acidilactici* and *Acremonium* cellulase. Means with different lowercases (a–d) among treatments at the same time point differed (*p* < 0.05; *n* = 4, bars indicate standard error of means).

The effects of additives and ensiling time on ferulic acid are presented in Figure 2. The figure revealed that the effects are significant throughout the ensiling time. At the initial phase of ensiling (3 d), the addition of *P. acidilactici* with commercial cellulase had a higher ferulic acid concentration (1155 mg/kg DM, *p* < 0.05), while no significant difference appeared among other groups. At 7 and 14 d, the highest concentration of ferulic acid was found in PA‐treated silage, implying that the *P. acidilactici* strain contributed to the dramatic increase in the concentration of ferulic acid. Meanwhile, the ferulic acid concentration of AC‐treated silage declined at 14 d of ensiling, and there was no difference between the Control and PA + AC‐treated silages. An increasing trend was observed in the AC and PA + AC groups in the mid‐stage of ensiling, and the highest ferulic acid content (1155 mg/kg DM, *p* < 0.05) was detected when the mixture of *P. acidilactici* and commercial cellulase was added compared to other silages. The concentration of ferulic acid had been on a plateau during the initial and mid ensiling phases of Control and PA treatments. As the fermentation advances to the end of ensiling, the trends remain similar for the ensiling time at 30 and 60 d except for Control silage where ferulic acid concentration declined. The PA + AC treatment had the highest ferulic acid content (1215 mg/kg DM, *p* < 0.05) when compared with Control, PA and AC groups after 60 d ensilage.

### Bacterial community composition and functional profiling in *C. korshinskii* silage

As shown in Table 3, the alpha diversity of *C. korshinskii* decreased after ensiling. Additives decreased the species richness (Chao 1, Observed species and ACE) compared with Control at the initial‐mid phase of ensiling (3–30 d), whereas PA + AC inoculated silage had the highest richness among the four groups at the end of the fermentation. PA inoculated silage had a lower Shannon value when compared with Control after 3 to 14 d of ensiling, whereas it had a higher Shannon value than the other three groups after 60 d of ensiling. The Shannon value in AC and PA + AC‐treated groups was lower than that of the Control from 3 to 7 d of ensiling, and lower than that of the PA inoculated group after 30 and 60 d of ensiling. In addition, the lowest Shannon value among the four treatments was observed in AC‐treated group after 30 and 60 d of ensiling.

**TABLE 3:** Bacterial alpha diversity of fresh and ensiled *C. korshinskii* as influenced by additives and ensiling period

Item	Fresh forage	Treatment (T)^a^	Ensiling time (E)					Mean	*SEM* ^b^	*p*‐value^c^		
			3	7	14	30	60			T	E	T × E
Chao1	306	Control	189^aA^	62.3^aB^	53.9^aC^	44.2^aD^	19.1^cE^	73.7^a^	0.279	0.001	0.001	0.001
		PA	55.8^cA^	23.1^cC^	47.0^bB^	21.1^cC^	14.0^dD^	32.2^c^				
		AC	101^bAB^	38.4^b^	41.5^bB^	28.0^bC^	26.1^bC^	46.9^b^				
		PA + AC	22.0^dB^	23.0^cB^	11.8^cD^	17.8^cC^	42.1^aA^	23.3^d^				
Shannon	5.56	Control	2.49^aA^	0.84^aB^	0.77^aBC^	0.67^bC^	0.66^bC^	1.09^a^	0.005	0.001	0.001	0.001
		PA	0.83^bA^	0.57^cB^	0.52^bB^	0.74^aA^	0.72^aA^	0.67^b^				
		AC	0.89^bA^	0.76^bB^	0.70^aC^	0.59^cD^	0.57^cD^	0.70^b^				
		PA + AC	0.80^bA^	0.76^bA^	0.75^aA^	0.65^bcB^	0.60^cB^	0.71^b^				
Observed species	273	Control	133^aA^	46.0^aB^	39.0^aB^	26.0^aC^	18.0^bC^	52.4^a^	0.384	0.001	0.001	0.001
		PA	29.0^cA^	16.0^cB^	9.00^cB^	13.0^bB^	12.0^cB^	15.7^c^				
		AC	70.0^bA^	26.0^bB^	16.0^bD^	15.0^bD^	20.0^bC^	29.4^b^				
		PA + AC	15.0^dB^	12.0^dB^	10.0^cB^	12.0^bB^	33.0^aA^	16.4^c^				
ACE	322	Control	210^aA^	81.0^aB^	65.4^aB^	64.8^aB^	28.0^bC^	89.8^a^	0.811	0.001	0.001	0.001
		PA	49.6^cA^	41.6^bA^	13.3^cB^	27.7^cAB^	26.8^bAB^	31.8^c^				
		AC	101^bA^	43.1^bB^	50.0^bB^	40.5^bBC^	29.5^bC^	52.8^b^				
		PA + AC	26.7^dB^	33.6^bB^	15.0^cC^	28.0^cB^	47.8^aA^	30.2^c^				

^a^ PA, *P. acidilactici*; AC, *Acremonium* cellulase; PA + PC, a combination of *P. acidilactici* and *Acremonium* cellulose; Means with different letters in the same row (A–E) or column (a–d) indicate a significant difference (*p* < 0.05).

^b^ *SEM*, standard error of the mean.

^c^ T, treatment; E, ensiling time (d); T × E, the interaction between treatment and ensiling time.

**FIGURE 3:**
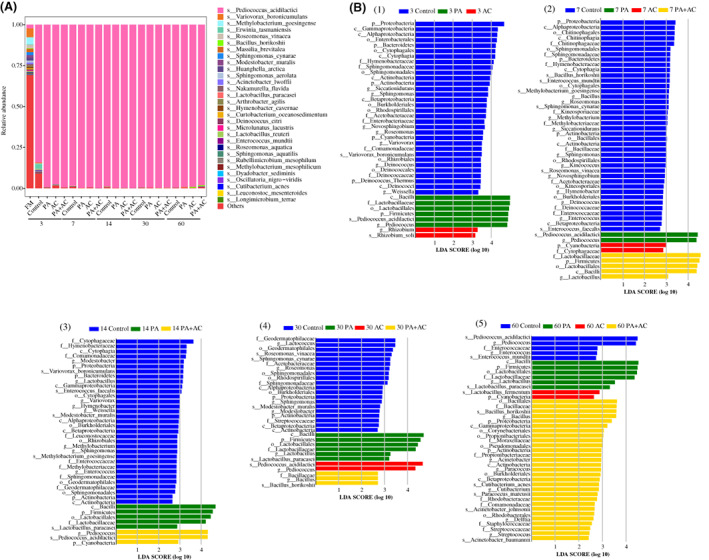
Bacterial community composition, differences and interactional networks of ensiled *C. korshinskii*. Treatment: Control, without additive; PA, *P. acidilactici*; AC, *Acremonium* cellulase; PA + AC, a combination of *P. acidilactici* and *Acremonium* cellulase. Arabic number indicating days of ensiling. (A) Comparison of microbiota compositions at species level of fresh and ensiled *C. korshinskii* as influenced by additives and ensiling period. (B) Comparison of the communities or species that have significant differences among different additive treatments and ensiling time using the LEfSe analysis.

**FIGURE 4:**
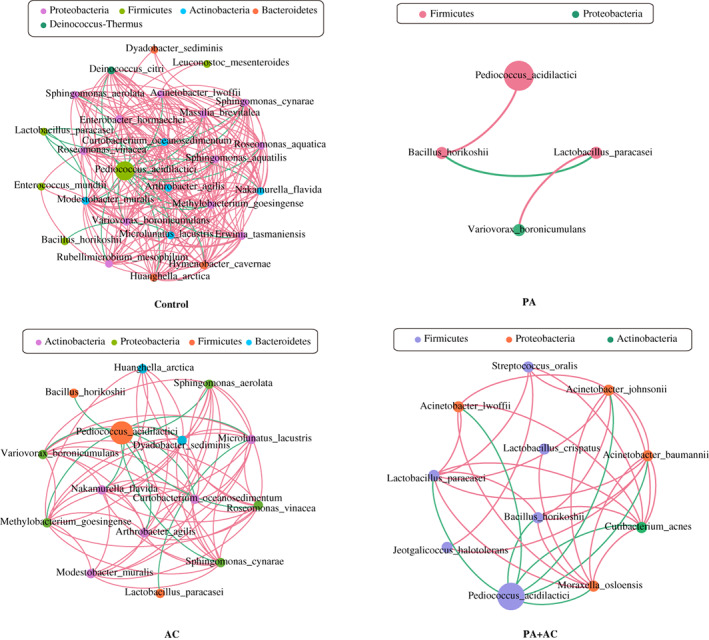
Comparison of interaction networks of the *C. korshinskii* silage microbiota. Node size is scaled based on the overall abundance of each taxon in the microbiota. Edge width is proportional to the strength of association between each metabolite‐phylotype pair (as measured by the correlation), red edge indicates positive correlations and green edge indicates negative corrections. Treatment: Control, without additive; PA, *P. acidilactici*; AC, *Acremonium* cellulase; PA + AC, a combination of *P. acidilactici* and *Acremonium* cellulase.

The composition of the bacterial community is presented in Figure 3A. The epiphytic microflora before ensiling was more complex, primarily comprised *Variovorax boronicumulans* (5.30%)*, Methylobacterium goesingense* (4.36%)*, P. acidilactici* (2.37%) and others (69.48%) at the species level. Regardless of pretreatment, the relative abundance of *P. acidilactici* dramatically increased and dominated the bacterial community of all the silages after 3 d. In addition to *P. acidilactici*, there were undesirable bacteria such as *Erwinia tasmaniensis* in the Control after 3 d of ensiling. As the ensiling period advanced from 7 to 60 d, *P. acidilactici* became the predominant species (>97%) in the *C. korshinskii* silage treated with or without additives. The linear discriminant analysis effect size (LEfSe) analysis was used to explore the differences in bacterial communities of the four *C. korshinskii* silage groups during the ensiling (Figure 3B). By comparing with the Control, the application of additives inhibited the growth of undesirable bacteria during the ensiling periods from 3 to 30 d. The relative abundance of *P. acidilactici* was significantly higher in PA inoculated silage after 3 and 7 d of ensiling, and *Lactobacillus paracasei* became the significantly abundant species in PA inoculated silage from 14 to 60 d of ensiling. In the AC‐treated silage, the relative abundance of *Rhizobium soli was* higher after 3 d of ensiling, and *P. acidilactici* and *Lactobacillus fermentum* were higher after 30 and 60 d of ensiling respectively. Interestingly, the relative abundance of undesirable bacteria such as *Bacillus horikoshii* was higher in PA + AC‐treated silage after 60 d of ensiling. The microbial networks of *C. korshinski* silage were calculated based on the 16S rRNA gene from the bacteria with a relative abundance greater than 0.001% (Figure 4). Simple microbial networks were observed in silages treated with additives, especially in PA inoculated silage. *P. acidilactici* was positively correlated with *B. horikoshii* in PA inoculated silage, while negatively correlated with *B. horikoshii* and other undesirable bacterial species in AC and PA + AC‐treated silage. The interaction between *P. acidilactici* and other bacterial species had complicated networks of bacterial interaction in the Control.

**FIGURE 5:**
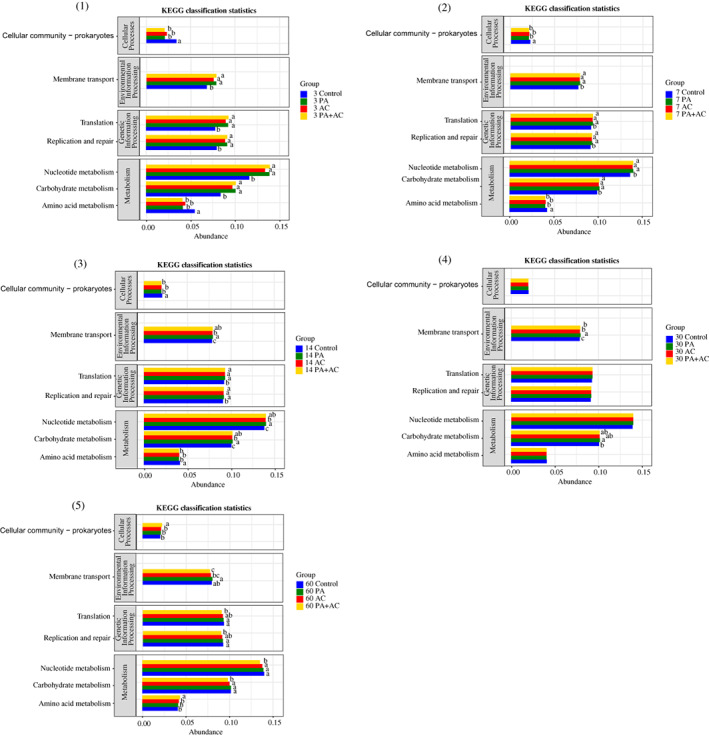
Level 2 KEGG orthologue gene of ensiled *C. korshinskii* as influenced by additives and ensiling period. Arabic number indicating days of ensiling. Treatment: Control, without additive; PA, *P. acidilactici*; AC, *Acremonium* cellulase; PA + AC, a combination of *P. acidilactici* and *Acremonium* cellulase. Functional prediction of bacterial changes in *C. korshinskii* after fermentation using PICRUSt2.

**FIGURE 6:**
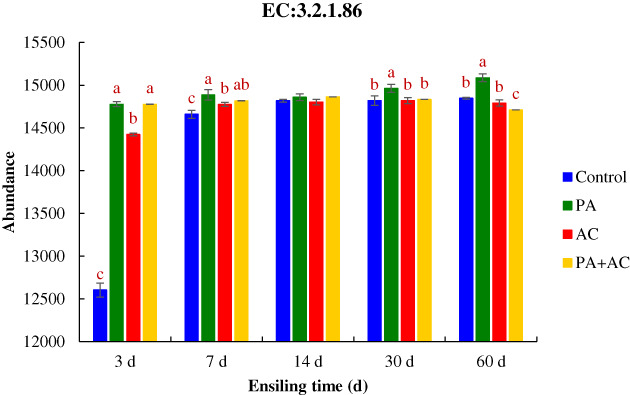
The abundance of 6‐phospho‐beta‐glucosidase (EC:3.2.1.86) of ensiled *C. korshinskii* as influenced by additives and ensiling period. Treatment: Control, without additive; PA, *P. acidilactici*; AC, *Acremonium* cellulase; PA + AC, a combination of *P. acidilactici* and *Acremonium* cellulase. Means with different lowercases (a–c) among treatments at the same time point differed (*p* < 0.05; *n* = 4, bars indicate standard error of means). EC:3.2.1.86 abundance prediction in *C. korshinskii* after fermentation using PICRUSt2.

The four Kyoto Encyclopedia of Genes and Genomes (KEGG) pathways were used to observe functional shifts, which were cellular processes, environmental information processing, genetic information processing and metabolism, among the bacterial community of the four treatments (Figure 5). The relative abundances of cellular community and amino acid metabolism in the Control were higher than those in additive‐treated groups at the initial stage of fermentation (3, 7 and 14 d), while the highest relative abundances were observed in the PA + AC at the 60 d of ensiling. In addition, the relative abundances of membrane transport, nucleotide metabolism and carbohydrate metabolism were lower in the Control than in the other three groups from 3 to 14 d of ensiling, while PA + AC inoculated silage showed the lowest relative abundances among the four treatments at the end of fermentation point. The 6‐phospho‐beta‐glucosidase selected from the enzyme classification (EC) database was used to describe the fibre degradation after fermentation (Figure 6). The abundance of 6‐phospho‐beta‐glucosidase was higher in PA‐inoculated silage than in Control and AC‐treated silage during the entire ensiling period except for 14 d of ensiling. In addition, there were no differences observed in 6‐phospho‐beta‐glucosidase abundance between PA and PA + AC treated silage after 3 and 7 d of ensiling, while the abundance of 6‐phospho‐beta‐glucosidase was higher in PA inoculated silage than in PA + AC‐treated silage after ensiling for 30 and 60 d.

### The effects of additives on enzymatic saccharification of *C. korshinskii* silage

**FIGURE 7:**
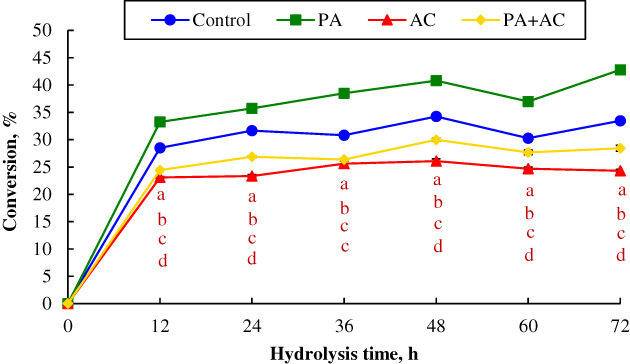
Cellulose conversion of ensiled *C. korshinskii* as influenced by additives and hydrolysis time. Treatment: Control, without additive; PA, *P. acidilactici*; AC, *Acremonium* cellulase; PA + AC, a combination of *P. acidilactici* and *Acremonium* cellulase. Means with different lowercases (a–d) among treatments at the same time point differed (*p* < 0.05; *n* = 4, bars indicate standard error of means).

The results of the enzymatic saccharification of the 60 d *C. korshinskii* silages are shown in Table 4 and Figure 7. The application of *P. acidilactici* had the highest glucose yield (*p* < 0.001) when compared with other treatments regardless of the hydrolysis times except at 24 h which had no significant difference with Control (Table 4). Meanwhile, the highest cellulose (*p* < 0.05) conversion was obtained in PA‐treated silage throughout the incubation period of the enzymatic saccharification, and the lowest cellulose conversion was observed in *Acremonium* cellulase‐treated silage except that the treatment was not different from the PA + AC group at 36 h (Figure 7). For the yield of xylose, the Control and PA‐treated silages were higher than AC and PA + AC groups (Table 4). Control silage exhibited the highest concentration of xylose throughout the incubation period, except at 36 h and no significant difference was found between Control and PA treatments at 48 h. The AC and PA + AC groups had similar xylose yields throughout the hydrolysis time.

**TABLE 4:** Glucose and xylose yields of ensiled *C. korshinskii* as influenced by additives and hydrolysis time

Item	Treatment (T)^a^	Hydrolysis time (H)						Mean	*SEM* ^b^	*p*‐value^c^		
		12	24	36	48	60	72			T	H	T × H
Glucose (g/kg DM)	Control	44.4^bD^	50.5^aB^	47.8^bC^	52.9^bA^	49.0^bC^	53.2^bA^	49.7^b^	0.110	<0.001	<0.001	<0.001
	PA	46.9^aC^	49.1^aC^	53.3^aB^	56.8^aA^	51.4^aB^	58.8^aA^	52.7^a^				
	AC	41.7^cB^	42.3^bB^	45.8^bA^	47.1^cA^	45.1^cA^	44.4^cB^	44.4^c^				
	PA + AC	38.4^dD^	42.8^bC^	42.5^cC^	46.1^cA^	43.7^cBC^	44.8^cAB^	43.1^d^				
Xylose (g/kg DM)	Control	6.27^aE^	8.54^aC^	8.09^bD^	9.83^aB^	12.8^aA^	9.88^aB^	9.23^a^	0.022	<0.001	<0.001	<0.001
	PA	5.81^bD^	7.84^bC^	8.99^aB^	10.1^aA^	7.93^bC^	8.96^bB^	8.27^b^				
	AC	4.76^cC^	5.56^cAB^	5.27^dB^	5.91^bA^	5.71^cAB^	6.08^cA^	5.55^c^				
	PA + AC	4.06^dC^	4.52^dC^	5.79^cB^	5.86^bB^	5.55^cB^	6.48^cA^	5.38^d^				

^a^ PA, *P. acidilactici*; AC, *Acremonium* cellulase; PA + PC, a combination of *P. acidilactici* and *Acremonium* cellulose; Means with different letters in the same row (A–E) or column (a–d) indicate a significant difference (*p* < 0.05).

^b^ *SEM*, standard error of the mean.

^c^ T, treatment; H, hydrolysis time (h); T × H, the interaction between treatment and hydrolysis time.

## DISCUSSION

*C. korshinskii* is widely planted in the arid and semi‐arid regions of China. However, its low WSC content and the high buffering capacity and lignocellulose content made it difficult to be ensiled (Ke et al., 2017; Li et al., 2021; Xu et al., 2006). Inoculating silage with LAB and cellulase was found to ensure good fermentation quality and improve bioconversion efficiency (Li, Ke, et al., 2020) because LAB can efficiently transform WSC into lactic acid and reduce silage pH to inhibit the growth of undesirable microorganisms at the initial stage of ensiling (Bai et al., 2021; Li, Zhang, et al., 2019). Moreover, some inoculants may have the ability to secrete cellulose‐related enzymes that help in hydrolysing the fibre structure of forages during ensiling (Chen et al., 2018; Li et al., 2021; Liu et al., 2019). Therefore, the addition of lactic acid‐producing cocci (*Pediococcus*) and cellulase as additives before ensiling was considered in the present study. Silages treated with PA were found to have the lowest WSC content compared to Control, AC and PA + AC‐treated silages, whereas the PA + AC group had higher lactic and acetic acid contents than the Control. Previous studies have shown that LAB strains can decline pH value by accelerating WSC transformation into lactic and acetic acids (Ding et al., 2019; Zhang et al., 2021). However, no differences in pH values were found between Control and PA treatments or between AC and PA + AC treatments after 60 d of ensiling. The most reasonable account was that *P. acidilactici* dominated the silage bacterial community at the early stage of fermentation (Bai et al., 2021; Yang et al., 2019). Cellulase increased lactic and acetic acid concentrations, which was consistent with the reports of Li et al. (2018) who reported that the addition of cellulase can promote the production of lactic and acetic acids through lignocellulose hydrolysis. The highest lactic and acetic acid concentrations in PA + AC‐treated silage could probably be due to the direct biodegradation of lignocellulose through the synergistic activity of *P. acidilactici* and cellulase (Oladosu et al., 2016; Li, Ke, et al., 2020). Generally, propionic acid is produced from secondary fermentation of clostridia by consuming lactic acid (Kung, 2008). Application of microbial inoculant and commercial cellulase decreased propionic acid concentration which inhibited secondary fermentation thereby preserving more nutrients excellently (Li, Ke, et al., 2020). In addition, the low DM losses (<23 g/kg DM) and pH values (<4.95) further proved that the additives are favourable to *C. korshinskii* ensilage in the present study. After 60 d of ensiling, *P. acidilactici* had a remarkably higher silage DM content because LAB inhibited the growth of spoilage microorganisms and their fermentation activities, which agrees with our previous study (Zhang et al., 2021). Proteolysis mainly resulted from plant proteases, but it could be inhibited when the pH has declined (Ke et al., 2017). The addition of *P. acidilactici* and *Acremonium* cellulase in the silages resulted in a greater reduction of NPN and NH_3_‐N contents after 60 d of ensiling. In addition, the reduction in NPN and NH_3_‐N contents after inoculation with *P. acidilactici* was mainly attributed to the decline in pH which occurred at the initial stage.

Many studies revealed that the addition of exogenous fibrolytic enzymes during ensiling can hydrolyse cell walls directly due to cellulase, glucanase and xylanase activities (Desta et al., 2016; Li, Ke, et al., 2020). The degradation of fibre (aNDF and ADF) was found in the AC‐treated group in the present study, which could be probably due to the coaction of various enzymes. Many studies have found that inoculation with LAB promoted the degradation of lignocellulose (Khota et al., 2016; Li, Ke, et al., 2020). Zhang et al. (2021) also revealed that *P. acidilactici* J17 strain which has a high antioxidant capacity enhanced the degradation of aNDF and ADF in alfalfa ensiled at two different DM contents. In this study, the degradation of aNDF, ADF and cellulose contents in PA‐treated silage could be attributed to the acid hydrolysis of the structural carbohydrates from the initial to the mid ensiling phase (Desta et al., 2016; Li, Ke, et al., 2020), or the production of cellulose‐related enzymes by *P. acidilactici* that accelerated the degradation of lignocellulose (Chen et al., 2018). Expectedly, the most effective degradation of aNDF, ADF and cellulose contents was found in PA + AC‐treated silage, which was attributed to the synergistic effect of cellulase and *P. acidilactici*. The synergistic action contributed by *P. acidilactici* was not only in utilizing fermentation substrates hydrolysed by *Acremonium* cellulase during ensiling but also in hydrolysing the structural carbohydrates through acidolysis or enzymolysis. Moreover, the inability to degrade lignin is the main limitation of animal digestibility and silage quality (Desta et al., 2016; Li, Ke, et al., 2020). In the current study, ADL exhibited a tendency of continuous decline during ensiling process and attained the lowest value at 30 d in PA‐treated silage. This further proved that *P. acidilactici* as a fermentation promoter, played a role in the fibre degradation of *C. korshinskii* at the early stage of ensiling. To further demonstrate the profile of lignocellulose degradation, the content of ferulic acid during ensiling was determined. Cellulose is covered by hemicellulose and lignin which limits the degradation of lignocellulose during ensiling (Pérez et al., 2002), reduces biomass digestibility and utilization by ruminants and hinders bioenergy production. Ferulic acid was released after the addition of *P. acidilactici* and *Acremonium* cellulase primarily through acidolysis and/or enzymatic hydrolysis which broke down the linkages of the ester bonds that binds the complex structure of cell wall polysaccharides. The silage treated with *P. acidilactici* exhibited a higher concentration of ferulic acid at the initial stage of fermentation, which further explained the reduction of aNDF and ADF contents of the silages. As expected, the highest content of ferulic acid was obtained in PA + AC‐treated silage after 60 d of ensiling, which is due to the synergistic effects of the additives.

The fermentation quality of silages with or without additives depends on the bacterial composition and changes during the ensiling time. Alpha diversity of *C. korshinskii* decreased after ensiling, and silage with additives had lower alpha diversity at the early stage of fermentation, which could be due to the dramatic decrease in pH. The acidic anaerobic environment after ensiling led to the modification of the bacterial community where most of the epiphytic bacteria disappeared due to their unadaptability to low pH (Dong et al., 2020; Méndez‐García et al., 2015; Zheng et al., 2017). Although the epiphytic LAB (*P. acidilactici*) in *C. korshinskii* before ensiling was the same as the added inoculant, the differences occurred due to the abundance and function. In the present study, the application of *P. acidilactici* decreased the relative abundance of *Erwinia tasmaniensis* and other epiphytic bacteria after 3 d of ensiling, and the *P. acidilactici* maintained the highest relative abundance throughout the entire fermentation period. This could be attributed to the adaptability as well as the rapid growth and multiplication of *P. acidilactici* which produces higher lactic acid that swiftly declined the pH to inhibit the growth of spoilage microorganisms throughout the fermentation (Bai et al., 2021; Yang et al., 2019). LEfSe analysis was used to further explore the differences in the bacterial community among the Control and additive treatments. The application of additives had weakened the growth of epiphytic competitors at the initial‐mid stage of ensiling. Subsequently, the competitiveness of some undesirable bacteria such as *B. horikoshi* resulted in the species increase in the PA + AC treatment at the end of ensiling. In the Control treatment, other species subsequently decreased while the epiphytic *P. acidilactici* abundance increased with the adaptation to the acidic environment at the end of fermentation. Our previous studies showed that a relatively simple network structure of bacterial interaction was attributed to a high fermentation quality, which led to a lower alpha diversity (Bai et al., 2021; Xu et al., 2020). In the current study, the additives simplified the network structures of the bacterial interaction after ensiling, especially PA‐treated silage showed the simplest bacterial interaction network structure which could be due to the coaction of epiphytic and exogenous *P. acidilactici* and resulted in a better fermentation quality.

The differences in fermentation quality among the different treatments despite having the same dominated bacteria species might be due to the varying degrees of microbial functions and degradation, as well as the transformation of fermentable substrates in the silages. To have a better understanding of regulating bacteria in silage fermentation, a functional predictive analysis of the metabolic pathway of bacteria was identified by PICRUSt. The metabolisms of nucleotide, carbohydrate and amino acid were closely related to silage fermentation (Bai et al., 2021; Xu et al., 2020). In the present study, nucleotide and carbohydrate metabolisms were lower in the Control at the initial process of silage fermentation and increased with the extension of the fermentation. According to Kilstrup et al. (2005) and Bai et al. (2021), the relative abundances of total LAB in the microbial community are related to the abundance of nucleotide and carbohydrate metabolic pathways. Based on the bacterial community compositions after ensiling, silages with high relative abundances of nucleotide and carbohydrate metabolisms had a higher relative abundance of *P. acidilactici*. And the relative abundance of carbohydrate metabolism was higher in AC than in the other treatments, which was in line with the higher WSC content. Generally, amino acids are the basic components of proteins and peptides. In this study, a higher relative abundance of amino acid metabolism was observed in the Control at 3 d, which suggests that *P. acidilactici* decreased the action of amino acid metabolism in other treatments mainly due to low pH that inhibits the action of protease. There were no differences among Control, PA and PA + AC‐treated silages in amino acid metabolism at the end of ensiling. However, lower CP content and higher NH_3_‐N and NPN concentrations were observed in the Control, which could be attributed to higher protein degradation and the accumulation of NH_3_‐N and NPN contents during the entire process of fermentation.

EC:3.2.1.86 can catalyse 6‐phosphate‐glucoside compounds to produce 6‐phospho‐glucose (Chen et al., 2018; Liu et al., 2014) and it was chosen to further explore the reason for lignocellulose degradation among different additives after ensiling. AC and PA + AC silages had a higher abundance of 6‐phospho‐beta‐glucosidase throughout the ensiling period (except 14 d), and PA‐treated group had the highest abundance of 6‐phospho‐beta‐glucosidase at the mid‐later fermentation process of the silage. This also explained the reason for the dramatic decline in aNDF and ADL in PA‐treated silage which is partly attributed to the role of 6‐phospho‐beta‐glucosidase and acid hydrolysis. The lowest aNDF and ADF observed in the PA + AC were also due to the synergistic effect of 6‐phospho‐beta‐glucosidase and *Acremonium* cellulase.

Lignocellulosic biomass provides an abundant cellulose and hemicellulose that can be converted into fermentable sugars during the anaerobic digestion stage. These sugars can further be used to produce bioproducts or biofuels (Fujii et al., 2009; Li, Ke, et al., 2020). However, the complex structure of lignocellulose results in the underutilization of the biomass by microbes or enzymes. Cellulose is mainly composed of glucose and macromolecular polysaccharides, but hemicellulose is a complex carbohydrate polymer that mainly consisted of glucose and xylose (Pérez et al., 2002). Due to the lower loss of sugar and production of by‐products, the enzymatic degradability of lignocellulose remains the best way to decrease environmental pollution (Shinozaki & Kitamoto, 2011). Pretreatment of forage before enzymatic saccharification is an effective method to improve cellulose conversion efficiency, mainly through the breakdown of the structural linkages (Desta et al., 2016; Li et al., 2018). In the present study, *C. korshinskii* with or without additives presented different yields of reducing sugars after hydrolysis. The PA‐treated silage had the highest glucose yield and cellulose conversion throughout the hydrolysis time. It could be due to acid hydrolysis and cellulose‐related enzymatic hydrolysis of *P. acidilactici* strain that modified the lignocellulosic structure in the initial process of ensiling (Chen et al., 2018; Desta et al., 2016). This also could be illustrated by the dramatic decline in aNDF and ADL at the early‐to‐mid stage of ensilage. According to Li, Ding, et al. (2019), inoculating corn stalk with ferulic acid esterase‐producing *L. plantarum* A1 had the highest glucose yield and cellulose convertibility during the enzymatic saccharification. When compared to Control and PA treatments, lower glucose and xylose yields, as well as lower cellulose conversion was observed in PA + AC treatment during the hydrolysis time. This could be due to the fact that forage enriched with exogenous *P. acidilactici* strain and cellulase resulted in lignocellulosic biomass (the most soluble structural polysaccharides portion) degradation during silage fermentation, and the remaining portion of the biomass became harder to be easily hydrolysed during enzymatic saccharification (Dehghani et al., 2012; Li, Ding, et al., 2019). Therefore, the highest ADL content was observed in PA + AC‐treated silage from early‐to‐mid and 60 d of fermentation time. Meanwhile, the highest decline in structural carbohydrates (aNDF and ADF) of PA + AC treatment during silage fermentation might have contributed to cellulose aggregation, thereby strengthening the fibre's resistance against enzyme action (Desta et al., 2016; Li, Ke, et al., 2020; Phitsuwan et al., 2016). In addition, silages with no additive exhibited higher xylose yield than AC and PA + AC treatments, it might be because of the highest contents of aNDF and ADF after 60 d of ensiling, aNDF and ADF were easier to be hydrolysed into xylose during enzymatic saccharification.

## CONCLUSION

After 60 d of ensiling *C. korshinskii*, all additives improved the fermentation quality as revealed by the concentrations of higher lactic and acetic acids as well as lower NPN and NH_3_‐N contents of the additives treated silages. Silage treated with PA + AC exhibited the lowest pH at the early‐to‐mid ensiling period. The addition of *P. acidilactici* and fibrolytic enzymes improved aNDF and ADL degradability and increased the concentration of ferulic acid, thereby making it an effective pretreatment for the hydrolysis of structural carbohydrates of *C. korshinskii* during silage fermentation. The additives modified the bacterial community of *C. korshinskii* silage and exhibited a relatively simple network structure of bacterial interaction (especially in PA‐treated silage). Additionally, higher 6‐phospho‐beta‐glucosidase abundance was also observed in PA‐treated silage due to the adaptability of *P. acidilactici* at the initial‐mid stage of ensiling. After enzymatic saccharification, PA‐treated silage had the highest lignocellulose conversion which is evidence of alteration of the lignocellulosic structure. Therefore, the present study provides a pretreatment method for preservation and conversion of the lignocellulosic residue of *C. korshinskii* through sole *P. acidilactici* treatment or in combination with *Acremoniuum* cellulase, thereby facilitating the utilization of the *C. korshinskii* biomass as feedstock for biofuel production.

## CONFLICT OF INTERESTS

The authors declare that they have no competing interests.

## Data Availability

Raw sequencing files and associated metadata have been deposited in NCBI's Sequence Read Archive (accession PRJNA827446), https://www.ncbi.nlm.nih.gov/sra.
